# Chemical carcinogenesis by 7,12-dimethylbenzanthracene in balb/c mice

**DOI:** 10.1186/1753-6561-7-S2-P46

**Published:** 2013-04-04

**Authors:** Marcello V Tedardi, Krishna D Oliveira, Gabriela U Avanzo, Marcelo MM Rangel, José L Avanzo, Heidge Fukumasu, Kurapati VK Rao, Idércio Luiz Sinhorini, Maria Lúcia Z Dagli

**Affiliations:** 1 Department of Pathology, School of Veterinary Medicine and Animal Science, University of Sao Paulo, Sao Paulo, SP, Brazil; 2 Department of Pathology, School of Animal Science and Food Engineering, University of Sao Paulo, Pirassununga, SP, Brazil

## Background

Experimental models of carcinogenesis have been used in cancer researches, mainly with radiation or chemicals like 7,12-dimethylbenzanthracene (DMBA) which is a polycyclic aromatic hydrocarbon. This chemical group contains the majority of carcinogens and requires biotransformation in the liver and mammary gland.

## Patients and methods

The carcinogen DMBA was administered by gavage to 70 Balb/c female mice, diluted in corn oil, at hebdomadary doses of 1 mg per animal for 1, 3, 6 or 9 weeks. Others 20 animals were used in the control group, who received only corn oil administration. Animals were weighed and monitored weekly until death, remaining animals were euthanized at age of 53 weeks. At necropsy, representative fragments of tumors were harvested, routinely processed for microscopy and classified agreeing with International Agency for Research on Cancer (IARC).

## Results

Control group hadn’t any behavioral or physical alterations. Tumors started being observed after 10 weeks of the beginning of DMBA protocol and the animals that received doses of 1 and 3mg survived more than 6 and 9mg groups. Were detected tumors in 67,14% of animals from DMBA groups and mammary tumors were the most common, counting 22 of them (31.43%). Cancer developed also in other primary sites: lungs (17.14%), lymphoid tissues (11,43%), stomach (7.14%) and skin (1.43%). Breast and gastric cancer were more frequents at higher doses and lung cancer in lower ones.

## Conclusion

It’s an effective and flexible carcinogenesis model that can be useful for studies for multiple cancers, especially in breast, gastric, lymphoid and lungs.

## Financial support

FAPESP.

